# Acute exacerbations in patients with progressive pulmonary fibrosis

**DOI:** 10.1183/23120541.00403-2024

**Published:** 2024-12-02

**Authors:** Michael Kreuter, Elizabeth A. Belloli, Elisabeth Bendstrup, Stefania Cerri, Kevin R. Flaherty, Shane Shapera, Jin Woo Song, Heiko Mueller, Klaus B. Rohr, Yasuhiro Kondoh, on behalf of the INBUILD trial investigators

**Affiliations:** 1 Center for Pulmonary Medicine, Department of Pneumology, Mainz University Medical Center and Pulmonary, Critical Care & Sleep Medicine, Marienhaus Clinic Mainz, Mainz, Germany; 2 Division of Pulmonary and Critical Care Medicine, University of Michigan, Ann Arbor, MI, USA; 3 Department of Respiratory Diseases and Allergy, Centre for Rare Lung Diseases, Aarhus University Hospital, Aarhus, Denmark; 4 Department of Clinical Medicine, Aarhus University, Aarhus, Denmark; 5 Center for Rare Lung Disease – Azienda Ospedaliero-Universitaria Policlinico di Modena, Modena, Italy; 6 Division of Respirology, University Health Network, University of Toronto, Toronto, ON, Canada; 7 Department of Pulmonary and Critical Care Medicine, Asan Medical Center, Seoul, South Korea; 8 Boehringer Ingelheim Pharma GmbH & Co. KG, Ingelheim am Rhein, Germany; 9 Boehringer Ingelheim International GmbH, Ingelheim am Rhein, Germany; 10 Department of Respiratory Medicine and Allergy, Tosei General Hospital, Japan; 11 For a list of the INBUILD trial investigators and their affiliations, see the Acknowledgements

## Abstract

**Background:**

Acute exacerbations of fibrosing interstitial lung diseases (ILDs) are associated with high mortality. We used prospective data from the INBUILD trial to investigate risk factors for acute exacerbations and the impact of these events in patients with progressive pulmonary fibrosis.

**Methods:**

Patients with progressive fibrosing ILDs other than idiopathic pulmonary fibrosis (IPF) were randomised to receive nintedanib or placebo. Associations between baseline characteristics and time to first acute exacerbation were assessed using pooled data from both treatment groups using Cox proportional hazard models, firstly univariable models and then a multivariable model using forward stepwise selection. The risk of death was estimated based on the Kaplan−Meier method.

**Results:**

Over a median follow-up of approximately 19 months, acute exacerbations were reported in 58 (8.7%) of 663 patients. In the risk factor analysis, the final model included diffusing capacity of the lung for carbon monoxide (*D*_LCO_) % predicted, treatment and age. Lower *D*_LCO_ % predicted was associated with an increased risk of acute exacerbation with a hazard ratio (HR) of 1.56 (95% CI 1.21–2.02) per 10 units lower (p<0.001). Age ≥65 years was associated with a numerically increased risk (HR 1.55, 95% CI 0.87–2.77; p=0.14). Treatment with nintedanib conferred a numerically reduced risk *versus* placebo (HR 0.60, 95% CI 0.35–1.02; p=0.06). The estimated risks of death ≤30 days and ≤90 days after an acute exacerbation were 19.0% (95% CI 8.9–29.2) and 32.0% (95% CI 19.7–44.2).

**Conclusions:**

Acute exacerbations of progressive pulmonary fibrosis may have similar risk factors and prognostic impact as acute exacerbations of IPF.

## Shareable abstract

**Data from the INBUILD trial suggest that acute exacerbations of progressive pulmonary fibrosis may have similar risk factors and prognostic impact as acute exacerbations of idiopathic pulmonary fibrosis** https://bit.ly/3LMA9ZI

## Introduction

Interstitial lung diseases (ILDs) are a large and heterogeneous group of disorders. Idiopathic pulmonary fibrosis (IPF) is an ILD of unknown cause that is always characterised by progressive pulmonary fibrosis [1]. A subset of patients with other fibrosing ILDs also develop progressive fibrosis, characterised by increasing radiological fibrosis, decline in lung function, worsening symptoms and high mortality [1–3]. The term “progressive fibrosing ILD” or “progressive pulmonary fibrosis” (PPF) is generally used to describe PPF in patients with a fibrosing ILD other than IPF. Various criteria have been proposed for the identification of PPF [1, 4–6]. All identify patients with progressive disease and poor outcomes [7, 8].

Acute exacerbations of fibrosing ILDs, characterised by acute deterioration in respiratory function and new widespread alveolar abnormality, are associated with high morbidity and mortality [9–12]. Acute exacerbations are a well-known feature of the natural history of IPF [10], but less is known about acute exacerbations of other ILDs. All of the available evidence comes from retrospective observational studies [11, 12–15], mostly conducted at single centres. To address this gap, we used prospective data from the INBUILD trial of nintedanib *versus* placebo [4, 16] to investigate risk factors for acute exacerbations and the prognostic impact of acute exacerbations in a broad population of patients with PPF.

## Materials and methods

### Patients

The design of the INBUILD trial (NCT02999178) has been published and the protocol is publicly available [4]. Briefly, patients had diffuse fibrosing ILD of >10% extent on high-resolution computed tomography (HRCT), forced vital capacity (FVC) ≥45% predicted and diffusing capacity of the lungs for carbon monoxide (*D*_LCO_) ≥30% to <80% predicted. Patients with IPF were excluded. Patients met ≥1 of the following criteria for ILD progression at any time within the prior 24 months, despite management deemed appropriate in clinical practice: a relative decline in FVC ≥10% predicted; a relative decline in FVC ≥5–<10% predicted and increased extent of fibrosis on HRCT; a relative decline in FVC ≥5–<10% predicted and worsened respiratory symptoms; worsened respiratory symptoms and increased extent of fibrosis on HRCT. Use of oral glucocorticoids at a dose of ≤20 mg·day^−1^ prednisone or equivalent was permitted. Patients taking azathioprine, cyclosporine, mycophenolate, tacrolimus, rituximab, cyclophosphamide or oral glucocorticoids >20 mg·day^−1^ prednisone or equivalent were not enrolled. Initiation of these medications was permitted after 6 months of the trial in patients with clinically significant deterioration of ILD or connective tissue disease, at the discretion of the investigator. The trial was carried out in compliance with the protocol, the principles of the Declaration of Helsinki and the Harmonised Tripartite Guideline for Good Clinical Practice from the International Conference on Harmonisation, and was approved by local authorities. All patients provided written informed consent before study entry.

### Trial design

Patients were randomised 1:1 to receive nintedanib 150 mg bid or placebo, stratified by fibrotic pattern on HRCT (usual interstitial pneumonia (UIP)-like fibrotic pattern or other fibrotic patterns) [4]. Patients continued to receive blinded randomised treatment until all patients had completed the post-treatment follow-up visit or entered the open-label extension study, INBUILD-ON (NCT03820726). The data available at this point comprised the data from the whole trial.

The criteria used for an acute exacerbation in the INBUILD trial were the same as the criteria for an acute exacerbation of IPF published by an international working group [10] except that they referred to a fibrosing ILD other than IPF. Thus, an acute exacerbation was defined as an event meeting all these criteria: acute worsening or development of dyspnoea (typically <1 month duration); computed tomography with new bilateral ground-glass opacity or consolidation superimposed on a background pattern consistent with fibrosing ILD; deterioration not fully explained by cardiac failure or fluid overload. Infection was not an exclusion criterion. Acute exacerbations were reported by the investigators as adverse events and were not adjudicated.

### Analyses

Analyses were conducted in patients who received ≥1 dose of trial drug and were conducted *post hoc*. The baseline characteristics of patients who had and did not have an acute exacerbation during the trial were assessed descriptively based on pooled data from both treatment groups. Comorbidity burden was assessed using the Charlson Comorbidity Index (CCI), which allocates scores based on age and the presence/absence of 19 comorbidities to provide a total score between 0 and 37 [17]. The seasonality of acute exacerbations was assessed based on the occurrence of the event in the Spring (Northern hemisphere: March–May; Southern hemisphere: September–November), Summer (Northern hemisphere: June–August; Southern hemisphere: December–February), Autumn (Northern hemisphere: September–November; Southern hemisphere: March–May) or Winter (Northern hemisphere: December–February; Southern hemisphere: June–August).

In a risk factor analysis, associations between patient characteristics at baseline and time to first acute exacerbation were assessed using pooled data from both treatment groups using Cox proportional hazard models. Associations were assessed first in univariable models and then in a multivariable model that employed forward stepwise selection. In the univariable models, p<0.05 was regarded as indicating a significant difference. To facilitate comparison with an analysis conducted using data from two similarly designed trials in patients with IPF [18], in the multivariable model, risk factors for acute exacerbation were identified by consecutively adding candidates into the model, selecting the covariate with the smallest p-value at each step, and selection was stopped when no further covariate achieved p<0.2. Candidate variables, selected based on prior associations with the risk of an acute exacerbation or mortality in patients with IPF or PPF, were age (<65 or ≥65 years), sex (male or female), race (Asian or non-Asian), body mass index (BMI) (<25, ≥25–<30 or ≥30 kg·m^−2^), smoking status (never or current/former), HRCT pattern (UIP-like fibrotic pattern or other fibrotic patterns), time since diagnosis of ILD (≤3 or >3 years), ILD diagnosis (unclassifiable idiopathic interstitial pneumonia (IIP), hypersensitivity pneumonitis, idiopathic nonspecific interstitial pneumonia (NSIP), autoimmune disease-related ILDs or other ILDs), FVC % predicted, *D*_LCO_ % predicted (corrected for haemoglobin), supplemental oxygen use (yes or no), use of corticosteroid or disease-modifying anti-rheumatic drug (DMARD) (yes or no), anti-acid medication use (yes or no) and trial medication (nintedanib or placebo). The Akaike information criterion (AIC) was calculated at each step to assess the goodness of fit of the statistical models. Hazard ratios and 95% Wald confidence intervals (CIs) were calculated to evaluate associations between each variable and time to first acute exacerbation.

The time from first acute exacerbation to hospitalisation and to death were analysed using pooled data from both treatment groups. The risks of hospitalisation and death were estimated based on the Kaplan−Meier method and 95% CIs were based on Greenwood's variance estimates.

## Results

### Patients

A total of 663 patients were treated in the INBUILD trial (332 with nintedanib and 331 with placebo). The baseline characteristics of these patients have been published [4]. In summary, mean (sd) age was 65.8 (9.8) years, 53.7% of patients were male, 73.6% were white, 51.0% were current or former smokers. Mean (sd) FVC was 69.0 (15.6) % predicted, mean (sd) *D*_LCO_ was 46.1 (13.6) % predicted. The ILD diagnoses were hypersensitivity pneumonitis (26.1%), autoimmune disease-related ILDs (25.6%), idiopathic NSIP (18.9%), unclassifiable IIP (17.2%) and other ILDs (12.2%).

### Baseline characteristics of patients with acute exacerbations during follow-up

Median follow-up during the INBUILD trial was approximately 19 months. Over this period, 58 patients (8.7%) had ≥1 acute exacerbation. Of these patients, 18 (31.0%) had hypersensitivity pneumonitis, 15 (25.9%) had unclassifiable IIP, 12 (20.7%) had autoimmune disease-related ILDs, 6 (10.3%) had idiopathic NSIP and 7 (12.1%) had other fibrosing ILDs. With regards to seasonality, 18 (31.0%), 19 (32.8%), 9 (15.5%) and 12 (20.7%) patients had their acute exacerbation in the Winter, Spring, Summer and Autumn, respectively.

Compared with the patients who did not have an acute exacerbation, the patients who had an acute exacerbation included a greater proportion of males (65.5% *versus* 52.6%) and patients with hypersensitivity pneumonitis (31.0% *versus* 25.6%) or unclassifiable IIP (25.9% *versus* 16.4%), a smaller proportion of patients with autoimmune disease-related ILDs (20.7% *versus* 26.1%) or idiopathic NSIP (10.3% *versus* 19.7%) and had a lower mean FVC (65.7% *versus* 69.3% predicted) and *D*_LCO_ (40.7% *versus* 46.6% predicted) at baseline (table 1). Mean (sd) CCI was 3.0 (1.4) and 2.8 (1.4) in patients who did and did not have an acute exacerbation, respectively. The inclusion criterion of a relative decline in FVC ≥10% predicted within the prior 24 months was met by a similar proportion of patients who did *versus* did not have an acute exacerbation (53.4% *versus* 49.9%, respectively).

**TABLE 1:** Baseline characteristics of patients who did and did not have an acute exacerbation during the INBUILD trial

	Had an acute exacerbation (n=58)	Did not have an acute exacerbation (n=605)
**Age, years, mean (sd)**	67.8 (9.1)	65.6 (9.8)
**Mal**e	38 (65.5)	318 (52.6)
**Body mass index, kg·m^−2^, mean (sd)**	27.4 (3.9)	28.4 (5.4)
**Current or former smoker**	31 (53.4)	307 (50.7)
**Race**	
White	43 (74.1)	445 (73.6)
Asian	15 (25.9)	148 (24.5)
Black/African-American	0	10 (1.7)
American Indian/Alaska Native/Native Hawaiian/ other Pacific Islander	0	1 (0.2)
**UIP-like fibrotic pattern on HRCT**	38 (65.5)	374 (61.8)
**FVC % predicted, mean (sd)**	65.7 (15.6)	69.3 (15.6)
***D*_LCO_ % predicted, mean (sd)**	40.7 (12.0)	46.6 (13.7)
**Time since diagnosis of ILD, years, mean (sd)**	3.5 (3.2)	3.8 (3.8)
**Use of DMARDs and/or corticosteroids**	35 (60.3)	348 (57.5)
**ILD diagnosis**		
Hypersensitivity pneumonitis	18 (31.0)	155 (25.6)
Autoimmune disease-related ILDs	12 (20.7)	158 (26.1)
Idiopathic NSIP	6 (10.3)	119 (19.7)
Unclassifiable IIP	15 (25.9)	99 (16.4)
Other fibrosing ILDs	7 (12.1)	74 (12.2)
**Inclusion criteria for ILD progression**		
Relative decline in FVC ≥10% predicted	31 (53.4)	302 (49.9)
Relative decline in FVC ≥5–<10% predicted and worsened respiratory symptoms	18 (31.0)	151 (25.0)
Relative decline in FVC ≥5–<10% predicted and increased extent of fibrosis on HRCT	6 (10.3)	73 (12.1)
Worsened respiratory symptoms and increased extent of fibrosis on HRCT	18 (31.0)	219 (36.2)

Data are presented as n (%) unless otherwise specified. Not all patients provided data for all variables. Autoimmune disease-related ILDs: RA-ILD, SSc-ILD, MCTD-ILD, autoimmune ILDs in “Other fibrosing ILDs” category of case report form. Other fibrosing ILDs: sarcoidosis, exposure-related ILDs and other terms in “Other fibrosing ILDs” category of case report form. UIP: usual interstitial pneumonia; HRCT: high-resolution computed tomography; FVC: forced vital capacity; *D*_LCO_: diffusing capacity of the lung for carbon monoxide; ILD: interstitial lung disease; NSIP: nonspecific interstitial pneumonia; DMARD: disease-modifying anti-rheumatic drug; IIP: idiopathic interstitial pneumonia.

### Risk factors for acute exacerbation

**FIGURE 1:**
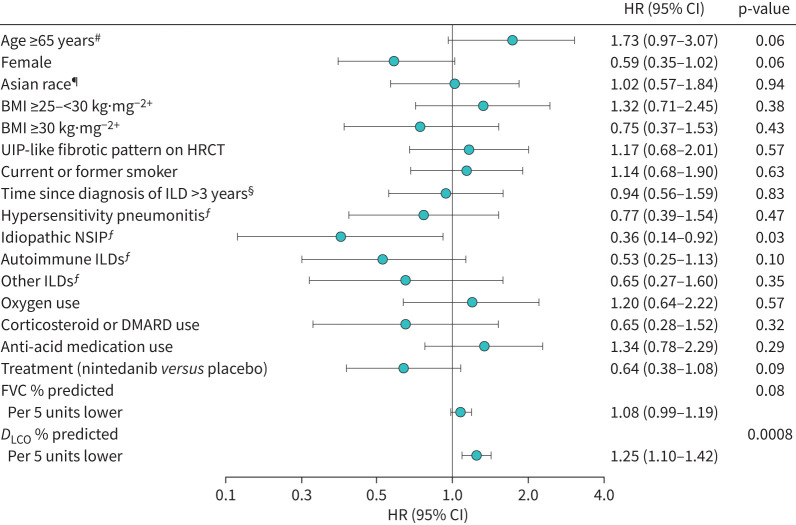
Associations between baseline characteristics and time to first acute exacerbation in the univariable model. HR, hazard ratio; CI: confidence interval; BMI: body mass index; UIP: usual interstitial pneumonia; HRCT: high-resolution computed tomography; ILD: interstitial lung disease; NSIP: nonspecific interstitial pneumonia; DMARD: disease-modifying anti-rheumatic drug; FVC: forced vital capacity; *D*_LCO_: diffusing capacity of the lung for carbon monoxide. ^#^: *versus* <65 years; ^¶^: *versus* non-Asian race; ^+^: *versus* <25 kg·mg^−2^; ^§^: *versus* ≤3 years; ^ƒ^: *versus* unclassifiable idiopathic interstitial pneumonia.

In the univariable models, lower *D*_LCO_ % predicted at baseline was significantly associated with an increased risk of acute exacerbation (HR 1.56, 95% CI 1.20–2.02 per 10 units lower) (figure 1). Lower FVC % predicted at baseline was associated with a numerically increased risk of acute exacerbation (HR 1.18, 95% CI 0.98–1.41 per 10 units lower) but statistical significance was not reached (p=0.08). A diagnosis of idiopathic NSIP was associated with a reduced risk of acute exacerbation compared with a diagnosis of unclassifiable IIP (figure 1). Age ≥65 years was associated with a numerically increased risk of acute exacerbation (HR 1.73, 95% CI 0.97–3.07; p=0.06) and female sex with a numerically reduced risk (HR 0.59, 95% CI 0.35–1.02; p=0.06) (figure 1).

**FIGURE 2:**
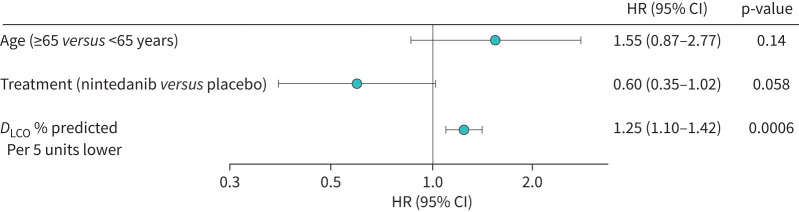
Associations between variables selected in the stepwise selection analysis and time to first acute exacerbation. The following variables were considered in the model: age, sex, race, body mass index, smoking status, high-resolution computed tomography pattern, time since diagnosis of interstitial lung disease (ILD), ILD diagnosis, forced vital capacity % predicted, diffusing capacity of the lung for carbon monoxide (*D*_LCO_) % predicted, supplemental oxygen use, corticosteroid or disease-modifying anti-rheumatic drug use, anti-acid medication use (all assessed at baseline) and treatment (nintedanib or placebo). Covariates that achieved p<0.2 in the stepwise selection procedure are shown. HR: hazard ratio; CI: confidence interval.

The stepwise variable selection in the multivariable model for associations between baseline characteristics and time to first acute exacerbation is shown in supplementary table 1. The final model included three variables: *D*_LCO_ % predicted, treatment (nintedanib *versus* placebo) and age (table 2). Lower *D*_LCO_ % predicted at baseline was associated with a significantly increased risk of acute exacerbation (HR 1.56, 95% CI 1.21–2.02 per 10 units lower) (figure 2) (p=0.0006). Age ≥65 years was associated with a numerically increased risk (HR 1.55, 95% CI 0.87–2.77; p=0.14). Treatment with nintedanib *versus* placebo was associated with a numerically reduced risk (HR 0.60, 95% CI 0.35–1.02; p=0.06) (figure 2). To assess whether the thresholds used for age and *D*_LCO_ % predicted had an impact on the associations observed, alternative thresholds were examined (>Q1 *versus* ≤Q1; >median *versus* ≤median; >Q3 *versus* ≤Q3; 2nd *versus* 1st quartile; 3rd *versus* 1st quartile; and 4th *versus* 1st quartile). Older age was significantly associated with an increased risk of acute exacerbation when the threshold was based on the median (>67 *versus* ≤67 years) (supplementary table 2). Lower *D*_LCO_ % predicted was significantly associated with an increased risk of acute exacerbation for all the thresholds examined (supplementary table 2).

**TABLE 2:** Summary of variables selected in the forward stepwise selection analysis

Step	Variable selected at the respective step	p-value	AIC for model after the respective step
1	*D*_LCO_ % predicted at baseline	0.0010	712.3
2	Treatment (nintedanib *versus* placebo)	0.039	710.0
3	Age	0.13	709.7

Lower AIC values indicate better performance of the statistical model. The following variables were considered for selection in the model: age (<65 or ≥65 years), sex (male or female), race (Asian or non-Asian), body mass index (<25, ≥25–<30, or ≥30 kg·m^−2^), smoking status (never or current/former), high-resolution computed tomography pattern (usual interstitial pneumonia-like fibrotic pattern or other fibrotic patterns), time since diagnosis of interstitial lung disease (ILD) (≤3 or >3 years), ILD diagnosis (unclassifiable idiopathic interstitial pneumonia, hypersensitivity pneumonitis, idiopathic nonspecific interstitial pneumonia, autoimmune disease-related ILDs or other ILDs), forced vital capacity % predicted, *D*_LCO_ % predicted, supplemental oxygen use (yes or no), corticosteroid or disease-modifying anti-rheumatic drug use (yes or no), anti-acid medication use (yes or no) (all assessed at baseline) and treatment (nintedanib or placebo). Covariates that achieved p<0.2 in the stepwise selection procedure are shown. AIC: Akaike information criterion; *D*_LCO_: diffusing capacity of the lung for carbon monoxide.

### Risk of hospitalisation and risk of death associated with acute exacerbation

**FIGURE 3:**
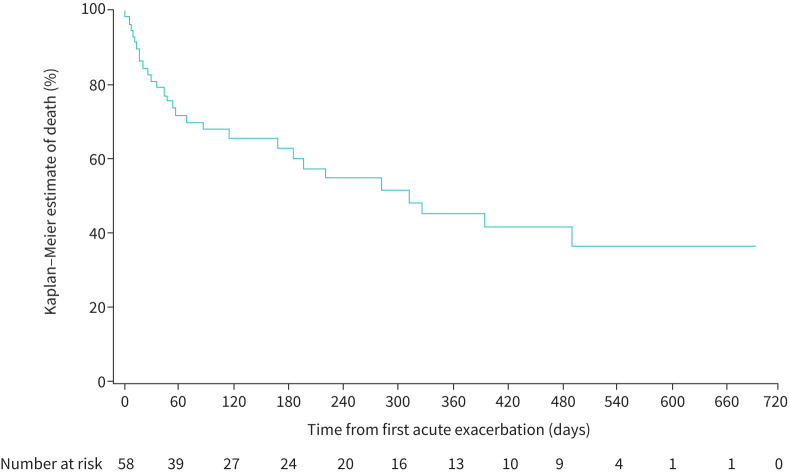
Time from first acute exacerbation to death.

The estimated risk (95% CI) of hospitalisation associated with the acute exacerbation or within 30 days following the event was 80.2% (69.7–90.7). The estimated risks (95% CI) of death ≤30, ≤60, ≤90 and ≤180 days after an acute exacerbation were 19.0% (8.9–29.2), 28.1% (16.4–39.8), 32.0% (19.7–44.2) and 37.0% (23.8–50.2) (figure 3). In patients with a UIP-like fibrotic pattern on HRCT, the estimated risk of death ≤180 days after an acute exacerbation was 39.3% (95% CI 22.9–55.7). In patients with other fibrotic patterns on HRCT, the estimated risk of death ≤180 days after an acute exacerbation was 33.3% (95% CI 10.5–56.2).

## Discussion

We used data from the INBUILD trial to investigate acute exacerbations in patients with PPF. To our knowledge, these are the first prospectively collected data assessing the frequency and impact of acute exacerbations in a multicentre study of patients with PPF.

While there is no established definition of an acute exacerbation in patients with PPF [19], studies of acute exacerbations in these patients have used definitions similar to those used to define acute exacerbations of IPF [10, 20]. In a recent consensus statement, experts agreed that acute exacerbations in patients with fibrosing ILDs are typically defined based on changes in symptoms and imaging, as per the definition of acute exacerbations of IPF [21]. In the American Thoracic Society/European Respiratory Society/Japanese Respiratory Society/Latin American Thoracic Association clinical practice guideline on PPF published in May 2022, the committee regarded the definition of an acute exacerbation of IPF as sufficient and did not propose an alternative, but acute exacerbation was not included in the criteria for ILD progression [1].

The reported frequency of acute exacerbations in patients with non-IPF ILDs varies widely [9, 11, 12, 14, 22], likely reflecting differences in the methodology used to define and capture acute exacerbations, as well as in the populations studied. In our analyses of data from the INBUILD trial, 8.7% of all patients had an acute exacerbation over a median follow-up of approximately 19 months. Among patients with IPF in the INPULSIS trials, 5.9% of all patients had an acute exacerbation over 52 weeks [23]. These data suggest that the risk of acute exacerbation is similar in patients with IPF and PPF, although it should be noted that the patients with PPF in the INBUILD trial had greater FVC impairment at baseline than patients with IPF in the INPULSIS trials (mean FVC 69% *versus* 80% predicted) [4, 23].

In our analyses, the risk of acute exacerbation was higher in patients who were aged ≥65 years or had lower *D*_LCO_ % predicted at baseline. Consistent with these findings, older age and greater impairment in *D*_LCO_ have been associated with risk of acute exacerbations in previous studies of patients with various ILDs [9, 11, 13, 14, 24, 25]. Unlike previous studies [9, 14, 26, 27], in our analysis, we did not observe a significant relationship between a UIP-like pattern on HRCT and the risk of acute exacerbation. This may reflect the inclusion criteria used in the INBUILD trial, which required that patients had reticular abnormality with traction bronchiectasis on HRCT as well as progression of lung fibrosis [4], or to differences in the risk of acute exacerbations between ILDs that are typically associated with a UIP-like pattern and those that are not.

We found that the risk of acute exacerbation was higher in patients with a lower FVC % predicted at baseline (by 18% per 10 units), although statistical significance was not reached. Prior studies have also shown that lower FVC % predicted is associated with an increased risk of acute exacerbations in patients with IPF and non-IPF ILDs [18, 28–31]. These findings suggest that treatments that slow lung function decline may reduce the risk of an acute exacerbation. Nintedanib inhibits processes fundamental to the progression of lung fibrosis [32] and has a consistent effect on slowing FVC decline across different types [33] and severities [34] of fibrosing ILD. Treatment with nintedanib has been associated with a reduced risk of acute exacerbation in patients with IPF in clinical trials and in observational studies [14, 18, 35, 36]. A multivariable analysis of data from the INPULSIS trials based on stepwise selection showed that patients with IPF who received nintedanib rather than placebo had a 34% reduction in the risk of acute exacerbation over 52 weeks [18]. In the current analysis of data from the INBUILD trial, treatment with nintedanib was associated with a numerically reduced risk of an acute exacerbation (HR 0.60, 95% CI 0.35–1.02; p=0.06).

Some studies have suggested that acute exacerbations of ILD may be more common in Winter than in other seasons, possibly due to an increased prevalence of respiratory infections during colder months [18, 37, 38]. In our analysis, the proportion of patients who had an acute exacerbation in Winter or Spring was two-fold greater than the proportion who had an acute exacerbation in Summer. Further research is needed into environmental conditions and other factors that may be triggers for acute exacerbations of ILD. Vaccination has been proposed as a measure to prevent acute exacerbations of ILD [19].

In the INBUILD trial, the risk of mortality in the 30 days following acute exacerbation was 19%. In the INPULSIS trials in patients with IPF, mortality within 30 days of an acute exacerbation was higher (21% in the nintedanib group and 40% in the placebo group) [18]. Previous studies have also found a higher risk of short-term mortality following acute exacerbations of IPF *versus* non-IPF ILDs [15, 24] although this has not been observed in all studies [11, 39]. Across studies, mortality following acute exacerbation in patients with IPF and other ILDs is very high, reflecting the need for effective treatments.

The strengths of our analyses include the collection of data in the setting of a clinical trial, with defined criteria for an acute exacerbation and standardised data collection. Limitations include the small number of patients who had an acute exacerbation, which limited the identification of risk factors and precluded conclusions being drawn on acute exacerbations in patients with particular ILD diagnoses. Stepwise selection procedures are established methods to select covariates that improve the fit of a statistical model. However, they have limitations, such as potential for bias and overfitting of the models [40]. No data were collected on whether patients had experienced acute exacerbations prior to the trial. There was no central review or adjudication of events. The number of patients with more than one acute exacerbation during the trial was too small to allow analyses of recurrent acute exacerbations. The effect of nintedanib on outcomes following an acute exacerbation could not be determined due to the low number of events. The CCI has limitations as a measure of comorbidity burden in patients with ILDs.

### Conclusions

In the INBUILD trial in patients with PPF, the risk of acute exacerbation was higher in patients who were aged ≥65 years, had a lower *D*_LCO_ % predicted or received placebo rather than nintedanib. Acute exacerbations were associated with a high risk of death in the subsequent 180 days. These data suggest that, as in patients with IPF, acute exacerbations have an important impact on outcomes in patients with PPF and may have similar risk factors. Further research is needed to inform the prevention and treatment of acute exacerbations of fibrosing ILDs.

A graphical abstract of the data presented in this manuscript is available at: www.globalmedcomms.com/respiratory/INBUILD_AcuteExacerbations.

## Supplementary material

10.1183/23120541.00403-2024.Supp1 **Please note:** supplementary material is not edited by the Editorial Office, and is uploaded as it has been supplied by the author.
Supplementary material 00403-2024.SUPPLEMENT

## Data Availability

To ensure independent interpretation of clinical study results and enable authors to fulfil their role and obligations under the International Committee of Medical Journal Editors criteria, Boehringer Ingelheim grants all authors access to relevant clinical study data. In adherence with the Boehringer Ingelheim Policy on Transparency and Publication of Clinical Study Data, scientific and medical researchers can request access to clinical study data, typically 1 year after the approval has been granted by major regulatory authorities or after termination of the development programme. Researchers should use https://vivli.org/ to request access to study data and visit www.mystudywindow.com/msw/datasharing for further information.
