# Supplementary material for: Acute exacerbations in patients with progressive pulmonary fibrosis
Source: ERJ Open Res. 2024 Dec 2;10(6):00403-2024. doi: 10.1183/23120541.00403-2024 (PMC11610068; doi:10.1183/23120541.00403-2024)
Supplement: Supplementary file 1 [file 00403-2024.SUPPLEMENT.pdf]

Acute exacerbations in patients with progressive pulmonary fibrosis

Supplementary material

Table S1. Forward stepwise variable selection using Cox proportional hazard model for associations between baseline characteristics and time to first acute exacerbation.

Variables	p-value for selection of variable in the respective step			
	**Step 1**	**Step 2**	**Step 3**	**Step 4**
**Treatment (nintedanib vs placebo)**	0.099	**0.039**	NA	NA
HRCT pattern	0.60	0.52	0.52	0.75
**Age**	0.071	0.094	**0.13**	NA
Sex	0.064	0.13	0.15	0.20
Race	0.98	0.86	0.91	0.95
BMI	0.21	0.22	0.19	0.26
Smoking status	0.68	0.88	0.96	0.91
Time since diagnosis of ILD	0.84	0.64	0.59	0.67
ILD diagnosis	0.18	0.25	0.23	0.34
FVC % predicted	0.089	0.72	0.71	0.56
**DLco % predicted**	**0.001**	NA	NA	NA
Oxygen use	0.59	0.66	0.66	0.58
Corticosteroid or DMARD use	0.30	0.37	0.34	0.33
Anti-acid medication use	0.27	0.56	0.49	0.51

In bold: Selected variable and p-value at the respective step ($p<0.2$). No variable was selected in step 4 as all p-values>0.2.

Note: p-values in Step 1 slightly differ from the univariable models shown in Figure 1 due to a reduced number of patients in this analysis as a result of missing baseline values: BMI (N=1), time since diagnosis of ILD (N=1), DLco % predicted (N=9).

Table S2. Associations between baseline characteristics and time to first acute exacerbation in the univariable model based on alternative thresholds for age and DLco % predicted.

**Variable**	**HR (95% CI)**	**P-value**
**Age (years)**		
>Q1 vs ≤Q1 (>60 vs ≤60)	1.07 (0.58, 1.97)	0.84
>median vs ≤median (>67 vs ≤67)	1.93 (1.13, 3.30)	0.02
>Q3 vs ≤Q3 (>73 vs ≤73)	1.27 (0.70, 2.30)	0.43
Q2 vs Q1 (>60–≤67 vs ≤60)	0.51 (0.21, 1.22)	0.05
Q3 vs Q1 (>67–≤73 vs ≤60)	1.58 (0.79, 3.15)	
Q4 vs Q1 (>73 vs ≤60)	1.26 (0.60, 2.64)	
**DLco % predicted**		
>Q1 vs ≤Q1 (>36.3 vs ≤36.3)	0.37 (0.22, 0.61)	<0.001
>median vs ≤median (>43.3 vs ≤43.3)	0.41 (0.24, 0.72)	0.002
>Q3 vs ≤Q3 (>53.6 vs ≤53.6)	0.46 (0.22, 0.98)	0.04
Q2 vs Q1 (>36.3–≤43.3 vs ≤36.3)	0.49 (0.26, 0.94)	0.001
Q3 vs Q1 (>43.3–≤53.6 vs ≤36.3)	0.33 (0.16, 0.69)	
Q4 vs Q1 (>53.6 vs ≤36.3)	0.27 (0.12, 0.61)	

Q, quartile.
